# Effect of different cement distribution in bilateral and unilateral Percutaneous vertebro plasty on the clinical efficacy of vertebral compression fractures

**DOI:** 10.1186/s12891-023-06997-4

**Published:** 2023-11-23

**Authors:** Abuduwupuer Haibier, Alimujiang Yusufu, Hang Lin, Aiben Kayierhan, Yimuran Abudukelimu, Alimujiang Aximu, Tuerhongjiang Abudurexiti

**Affiliations:** 1 Department of Orthopedics of Trauma, Sixth Afliated Hospital of Xinjiang Medical University, Orthopaedic Hospital of Xinjiang Uygur Autonomous Region, No.39 Wuxing Road, Urumqi, People’s Republic of China; 2 Xinjiang Medical University, Uygur Autonomous Region, Urumqi, Xinjiang People’s Republic of China

**Keywords:** Osteoporotic vertebral compression fracture, Percutaneous vertebro plasty, Bone cement, Bone cement distribution

## Abstract

**Background:**

The ramifications of osteoporotic fractures and their subsequent complications are becoming progressively detrimental for the elderly population. This study evaluates the clinical ramifications of postoperative bone cement distribution in patients with osteoporotic vertebral compression fractures (OVCF) who underwent both bilateral and unilateral Percutaneous Vertebroplasty (PVP).

**Objective:**

The research aims to discern the influence of bone cement distribution on the clinical outcomes of both bilateral and unilateral Percutaneous Vertebroplasty. The overarching intention is to foster efficacious preventive and therapeutic strategies to mitigate postoperative vertebral fractures and thereby enhance surgical outcomes.

**Methods:**

A comprehensive evaluation was undertaken on 139 patients who received either bilateral or unilateral PVP in our institution between January 2018 and March 2022. These patients were systematically classified into three distinct groups: unilateral PVP (*n* = 87), bilateral PVP with a connected modality (*n* = 29), and bilateral PVP with a disconnected modality (*n* = 23). Several operational metrics were juxtaposed across these cohorts, encapsulating operative duration, aggregate hospital expenses, bone cement administration metrics, VAS (Visual Analogue Scale) scores, ODI (Oswestry Disability Index) scores relative to lumbar discomfort, postoperative vertebral height restitution rates, and the status of the traumatized and adjacent vertebral bodies. Preliminary findings indicated that the VAS scores for the January and December cohorts were considerably reduced compared to the unilateral PVP group (*P* = 0.015, 0.032). Furthermore, the recurrence of fractures in the affected and adjacent vertebral structures was more pronounced in the unilateral PVP cohort compared to the bilateral PVP cohorts. The duration of the procedure (*P* = 0.000) and the overall hospitalization expenses for the unilateral PVP group were markedly lesser than for both the connected and disconnected bilateral PVP groups, a difference that was statistically significant (*P* = 0.015, *P* = 0.024, respectively). Nevertheless, other parameters, such as the volume of cement infused, incidence of cement spillage, ODI scores for lumbar discomfort, post-surgical vertebral height restitution rate, localized vertebral kyphosis, and the alignment of cement and endplate, did not exhibit significant statistical deviations (*P* > 0.05).

**Conclusion:**

In juxtaposition with unilateral PVP, the employment of bilateral PVP exhibits enhanced long-term prognostic outcomes for patients afflicted with vertebral compression fractures. Notably, bilateral PVP significantly curtails the prevalence of subsequent vertebral injuries. Conversely, the unilateral PVP cohort is distinguished by its abbreviated operational duration, minimal invasiveness, and reduced overall hospitalization expenditures, conferring it with substantial clinical applicability and merit.

## Introduction

In light of the escalating demographic transition towards an older population, there has been a marked uptick in the incidence of osteoporotic vertebral compression fractures, which are becoming increasingly prevalent on an annual basis. This trend presents a profound impact on the quality of life of the affected patients, amplifying the exigency of medical attention and interventions [1, 2]. Indeed, osteoporotic vertebral compression fractures rank as one of the paramount complications associated with osteoporosis [3]. Such fractures not only manifest as chronic back pain and localized vertebral kyphosis, diminishing the patient's quality of life, but also augment the potentiality for disability and, in some instances, elevate mortality risks [4]. Within the confines of the United States alone, it is posited that there emerge approximately 140,000 new cases of these fractures annually [5].

The advent of Percutaneous vertebroplasty, entailing the minimally invasive administration of polymethylmethacrylate bone cement, offers a beacon of hope by bestowing immediate alleviation from pain and fortifying the fractured vertebrae [6]. Due to its commendable safety credentials and consistently efficacious short-term outcomes, this modality has ascended to be the predominant therapeutic strategy for osteoporotic vertebral compression fractures in recent chronological milestones [7, 8]. Yet, despite evidential affirmations suggesting that symmetrical cement distribution is pivotal in mitigating the recurrence of postoperative vertebral fractures and fostering a favorable prognosis, achieving this desired cement equilibrium during Percutaneous vertebroplasty still poses a conundrum for many medical practitioners [1, 2].

To this end, a lacuna persists in the current literature, with a paucity of comprehensive studies elucidating the ramifications of cement distribution vis-à-vis postoperative outcomes following Percutaneous vertebroplasty. In addressing this research gap, the present investigation conducted a retrospective scrutiny of clinical data amassed from patients diagnosed with osteoporotic vertebral compression fractures and subsequently treated at the Sixth Affiliated Hospital of Xinjiang Medical University spanning from January 2018 to March 2022. Leveraging ANOVA, the study delineated the correlation between bone cement distribution and postoperative outcomes post both bilateral and unilateral Percutaneous vertebroplasty. The overarching aspiration of this endeavor is to proffer enhanced preventive and therapeutic paradigms, with the dual objectives of obviating postoperative vertebral re-fractures and refining surgical efficacies.

## Objects and methods subjects and methods

### Design

Retrospective comparative trial.

### Time and location

The study was conducted at the Department of Spine Surgery, Sixth Affiliated Hospital of Xinjiang Medical University, spanning from January 2018 to March 2022.

### Inclusion criteria

(i) Patients who, for the first time, sought intervention at the Department of Spine Surgery of the Sixth Affiliated Hospital of Xinjiang Medical University between January 2018 and March 2022 to undergo either bilateral or unilateral Percutaneous vertebroplasty. (ii) Fresh fractures complemented by osteoporotic alterations, corroborated via radiographic examinations encompassing X-ray, CT, MRI, and bone densitometry assessments. (iii) All enrolled participants furnished informed consent, duly endorsed by the Ethics Committee of the Sixth Affiliated Hospital of Xinjiang Medical University. (iv) The affliction was confined to a maximum of two fractured vertebrae. (v) An exhaustive follow-up record spanning a minimum of 12 months post-intervention.

### Exclusion criteria

(i) Manifestations of preoperative nerve root injury. (ii) Demonstrated hypersensitivity to bone cement constituents. (iii) Severe neurological, psychiatric disorders, or any foundational maladies rendering the patient incapable of participating in pain evaluations. (iv) Pathological fractures stemming from malignancies or infections. (v) Patients with antecedent spinal surgeries. (vi) Body mass index surpassing 35 kg/m^2^.

### Classification of bone cement distribution

Based on anteroposterior radiographic assessments (either X-ray or CT) of the vertebral column, bone cement distribution was demarcated as: (i) **Unilateral PVP:** Connection group, inferred from frontal X-ray or CT imagery of the vertebral body. (ii) **Bilateral PVP:** Categorized into two subsets—bone cement connection group and bone cement non-connection group.

**Fig. 1:**
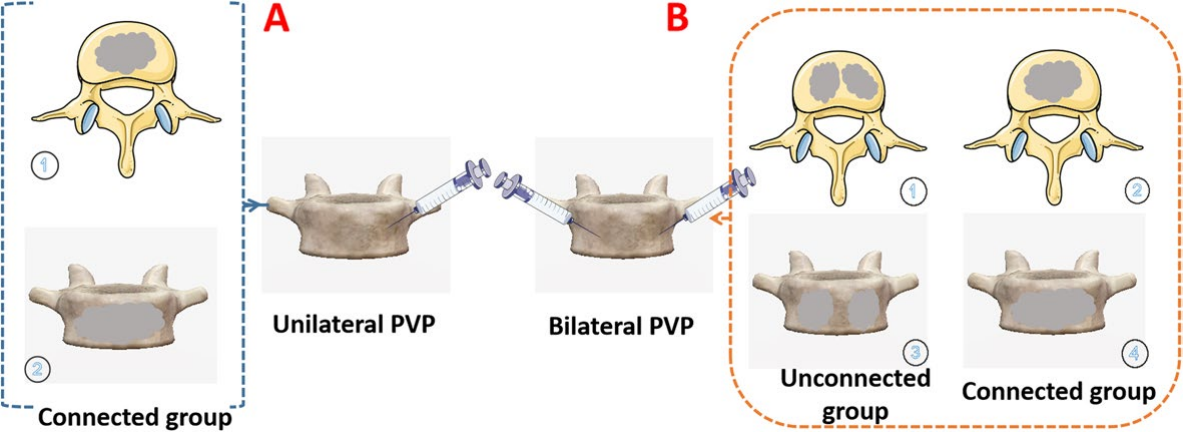
Type of bone cement distribution. Section A represents the connection subset of the unilateral PVP group, with Parts ①② epitomizing the connection classification. Conversely, Section B symbolizes the bilateral PVP category, wherein Parts ①③ depict the non-connected bilateral PVP faction, and Parts ②④ embody the connected bilateral PVP faction

Subsequent to evaluating the cement's locational distribution, its dispersion across the two surgical interventions was segregated into three distinct categories, elucidated in Fig. 1:

- **Group A:** Unilateral PVP connection group (Refer to Fig. 1, Parts A ①②).
- **Group B:** Bilateral PVP with connectomic attributes (Refer to Fig. 1, Parts B ②④).
- **Group C:** Bilateral PVP devoid of connections (Refer to Fig. 1, Parts B ①③).

### Surgical method

#### Preoperative preparation

Prior to the vertebroplasty procedure, comprehensive diagnostics including X-rays, CT scans, and MRI were administered. Additionally, routine hematological evaluations, hepatic and renal function tests, serum bone metabolic indices, and bone densitometry assessments were executed.

#### PVP surgery method

1. ***Unilateral PVP:*** Patients were oriented in a prone position. The targeted vertebral body was precisely identified using C-arm fluoroscopic guidance. Following this, standardized disinfection and draping procedures were executed. Local anesthesia was achieved via infiltration with a 2% lidocaine hydrochloride solution (Tianjin Jinyao Group Hubei Tianyao Pharmaceutical Co., Ltd., Specification: 5 mL: 0.1 mg, State Drug Quantification: H20133209). Employing a unilateral puncture approach, bone cement was subsequently prepared for placement. The positioning of the puncture needle was meticulously refined under C-arm guidance. Thereafter, a bone hammer was utilized to delicately introduce the bone cement-loaded needle into the vertebral pedicle. The needle's placement was cross-verified using C-arm assistance, ensuring its insertion into the proximal third of the vertebral body midline. Upon confirming the precise positioning, the needle core was retracted, and polymethylmethacrylate bone cement was prepared for instillation. Utilizing C-arm monitoring, a volume ranging between 1-9 mL of bone cement was methodically infused into the vertebral body. Constant vigilance was maintained to ensure appropriate cement distribution. Following cement solidification, the puncture needle was safely extracted, and the access point was secured with a sterile dressing. Immediate post-operative radiographs were captured. Patients were then carefully transported to their respective wards on a stretcher. Post-operative recovery allowed for limited movement, facilitated by a lumbar brace, 6–8 h post-surgery.
2. ***Bilateral PVP:*** This procedure closely mirrored the unilateral technique. With anteroposterior fluoroscopic monitoring, the needle's silhouette exhibited a "bull's-eye" configuration. This needle was cautiously advanced, aiming for a central puncture route from the pedicle into the vertebral body. Under continuous fluoroscopy, bone cement was gradually introduced, with utmost care taken to preemptively circumvent any potential cement leakage. Instrument specifications can be referred to in Tables 1, 2 and 3.

**Table 1:** Material characteristics of the implant

Product Name	Bone Cement Bone Cement (REF66055104)
Manufacturers	Heraeus Medical GmbH (Heraeus Medical)
Specification Model	OSTEOPAL V^Ⓡ^
Approval Number	LOT 61185327
Structure and composition/main constituents	Osteopal V is a fast setting, radiation-impermeable bone cement for filling and stabilizing vertebrae. The product contains a powder and a liquid. The powder consists of methyl acrylate-methyl methacrylate polymer, zirconium dioxide, benzoyl peroxide, and copper chlorophyll (E141).The liquid consists of methyl methacrylate, N,N-dimethyl-p-toluidine, hydroquinone, and copper chlorophyllin (E141)
Scope of application/intended use	Osteopal V is indicated for filling and stabilization of the vertebral body: relief and elimination of pain in vertebral compression fractures, relief and elimination of pain in vertebral tumors (metastatic or bone marrow cancer), symptomatic vertebral hemangiomas
Biocompatibility	Good
Adverse reactions	May cause adverse effects such as hypotension, hypoxemia, arrhythmia, cardiac arrest, cardiopulmonary dysfunction, and even death

**Table 2:** Complete set of surgical instruments for vertebroplasty

Device name	Spiral Propeller
Specification	20 ml
Model	Type 201
Registration certificate number	National Machinery Note approved 20,153,040,284
Production companies	Shandong Guanlong Medical Supplies Co

**Table 3:** puncture needle

Device name	Puncture Needle
Specification	2.5 × 130
Model	GC-01
Registration certificate number	RuMechanicsNotePermission20142140147
Production companies	Shandong Guanlong Medical Supplies Co

#### Postoperative management

Upon completion of the surgical procedure, patients were mandated a strict recumbent period of 6–8 h. During mobilization, their torso was securely encompassed with a thoraco-lumbar support belt to facilitate ambulation.

For optimal bone health and to fortify the surgical intervention, a daily oral regimen of calcium carbonate tablets (600 mg/day) and vitamin D was strongly endorsed, to be continued for a minimum duration of one year post-operation.

In addressing the osteoporotic etiology, patients across all three cohorts were administered an intravenous dose of zoledronic acid (Yangtze River Pharmaceutical Group Sichuan Hailong Pharmaceutical Co., Ltd., Specification: 100 ml: 5 mg, State Drug Quantifier: H20183098) on the immediate subsequent day post-vertebroplasty. Prior to its instillation, an intravenous hydration protocol comprising of a 500 mL drip of 0.9% sodium chloride solution (Sichuan Keren Pharmaceutical Co., Ltd., Specification: 500 ml, State Drug Administration: H51021158) was initiated to optimize renal function and overall hydration.

To further augment postoperative recuperation, patients were advised to maintain a heightened fluid intake. Additionally, they were educated on the significance of meticulously documenting any untoward symptoms or adverse reactions—emphasizing the onset, severity, and duration. In the event of a postoperative pyrexia surpassing 38.5℃, the provision of oral non-steroidal anti-inflammatory agents was recommended to temper the febrile response. Moreover, adjunctive therapeutic measures, such as physical cooling and enhanced hydration, were implemented as symptomatic relief.

One week postoperatively, a rigorous radiographic evaluation encompassing both X-ray and computed tomography (CT) scans was conducted. This imaging suite was paramount in delineating the exact loci and distribution of the infused bone cement and in assessing the post-surgical vertebral height restoration.

### Postoperative index evaluation

**Fig. 2:**
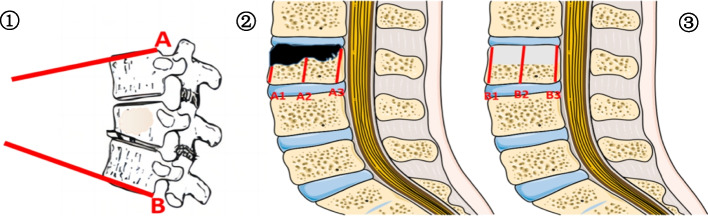
Measurement of postoperative imaging parameters. Figure ①: Depicts two parallel lines drawn with respect to the fractured vertebra. Line A is aligned with the upper end plate of the vertebral body immediately superior to the fracture site, while Line B corresponds to the lower end plate of the vertebral body directly inferior to the fracture. Figure ②: Illustrates the structural facets of a preoperative vertebra. Specifically: A1 delineates the posterior edge, A2 represents the height corresponding to the preoperative fracture, A3 demarcates the anterior edge. Figure ③: Highlights the anatomical features of a typical vertebral body: B1 defines the posterior edge, B2 indicates the overall height of the vertebral body, and B3 pinpoints the anterior edge

1. **Local Kyphosis Angle (LKA)**: Let's consider line A as the line parallel to the upper endplate of the vertebra immediately superior to the fractured vertebra. Similarly, line B is parallel to the lower endplate of the vertebra immediately inferior to the fractured vertebra. The local kyphosis angle (lka) is the angle subtended between lines A and B, as depicted in Fig. 2①.
2. **Postoperative imaging parameters were measured** **:** **Estimated Original Vertebral Body Height (EOH)**: Defined as the sum of the height lost due to injury and the height prior to treatment. Methodologically, EOH is derived by considering the mean height of the intact vertebral bodies situated immediately superior and inferior to the compromised vertebra.
**Preoperative Fractured Vertebral Body Height (PFH, denoted as A2)**: This is the disparity between the projected vertebral height and the actual height lost post-injury. Quantitatively, PFH is extracted from the mean value between the posterior (A1) and anterior (A3) boundaries of the vertebral body, as graphically elucidated in Fig. 2.
**Postoperative Restored Vertebral Body Height (PRH, denoted as B2)**: Representing the difference between the post-treatment and pre-treatment vertebral heights, PRH is mathematically the average of the posterior (B1) and anterior (B3) edges of the vertebra (Refer to Fig. 2 for a visual representation).
**Height Restoration Rate (HRR)**: Serving as an indicative metric of surgical efficacy, HRR quantifies the proportion of vertebral height restoration, postoperatively. The formulaic representation for the same is: HRR = (PRH-PFH) / EOH × 100%. (Fig. 2 ② ③) [9].
**Local Kyphosis Angle (LKA)**: This parameter delineates the angular measure formed by lines orientated parallel to the upper end plate of the vertebra immediately superior and the lower end plate of the vertebra immediately inferior to the fracture site.
3. **Bone Cement Leakage**: This pertains to instances where bone cement, post-injection, extends beyond the intended vertebral body, infiltrating into adjacent tissues during the PVP procedure.
4. **Adjacent Vertebral Fracture**: This is characterized by the emergence of fractures in the two vertebrae immediately adjacent to the originally injured vertebra post-intervention.
5. **Re-fracture Evaluation of the Injured Vertebrae**: Post-PVP, if patients exhibit renewed lumbar discomfort coupled with a clinical finding of positive percussion pain localized either to the vertebral spine or spinous process, it warrants further investigation. Subsequent lumbar spine radiographs suggesting vertebral body compression fracture at the percussion pain site, corroborated by magnetic resonance imaging (MRI), confirm the diagnosis of re-fracture.
6. **Total Hospitalization Expenditure**: This is a comprehensive financial metric reflecting the entirety of the patient's expenditure from admission to discharge.

### General information

We conducted a comprehensive retrospective analysis of medical records belonging to 139 patients who underwent bilateral Percutaneous Vertebroplasty (PVP) and unilateral PVP at our institution between January 2018 and March 2022. Based on the bone cement distribution pattern observed during these procedures, patients were stratified into three distinct cohorts:

1. Unilateral PVP group (*n* = 87), Unilateral PVP group (*n* = 87),
2. Bilateral PVP-connected group (*n* = 29), and
3. Bililateral PVP-unconnected group (*n* = 23).

An analysis of variance (ANOVA) was employed to discern the influence of bone cement distribution on post-procedural outcomes following both bilateral and unilateral PVP. The demographic and clinical attributes assessed across the three groups encompassed patient gender, age, body mass index (BMI), BMD T value, PVP stage, and relevant medical history.

### Observed indicators

**(1) Primary Outcome Measures:** The following parameters were meticulously evaluated at specified timepoints:

- Vertebral Analog Scale (VAS) scores for low back pain at preoperative, 1-week, 1-month, and 1-year post-operative intervals,
- Oswestry Disability Index (ODI) scores before surgery and 1 week, 1 month, and 1 year after the procedure,
- Vertebral body height recovery rate and local posterior kyphotic angle, assessed 1 week and 1 year postoperatively,
- Incidence of re-fracture in both the operated and adjacent vertebrae, and
- Positional analysis of bone cement vis-à-vis the end plates.

The VAS is a diagnostic tool with a score ranging from 0 (indicating no pain) to 10 (indicating maximum pain). The ODI was leveraged to gauge improvements in daily functional capacity, evaluating various domains such as pain intensity, capacity to lift, walking efficiency, sleep quality, and social life interactions [10]. An ODI score of 0% denotes minimal disability, whereas a score of 100% indicates profound disability, possibly necessitating bed rest or representing exaggerated symptomatology.

**(2) Secondary Outcome Measures:** These included the time elapsed to surgery, the volume of bone cement utilized, and instances of cement leakage.

#### Statistical methods

All analytical operations were executed using the SPSS 26.0 software suite. The acquired data, collected bilaterally, were quantitatively represented as mean ± standard deviation, while categorical data were conveyed as proportions or rates. Subsequent to normality tests, ANOVA was employed for inter-group comparisons if data distributions were found to be normal. Non-normal data distributions necessitated the use of the Kruskal–Wallis H test. For binary procedural comparisons, the two independent samples t-test was selected. Rate comparisons across multiple cohorts were facilitated using the χ2 test. A p-value less than 0.05 was deemed indicative of statistically significant disparities.

## Results

### Analysis of the number of participants

A cohort of 139 patients, each undergoing bilateral Percutaneous Vertebroplasty (PVP) and unilateral PVP for the initial time, was incorporated into the study. Based on the mode of bone cement distribution during these procedures, the patients were systematically segmented into three groups:

- 87 patients in the Unilateral PVP category,
- 29 patients in the Bilateral PVP-Connected category, and
- 23 patients in the Bilateral PVP-Unconnected category.

All participants were comprehensively included in the outcomes assessment with no data exclusion.

### Experimental flow chart

**Fig. 3:**
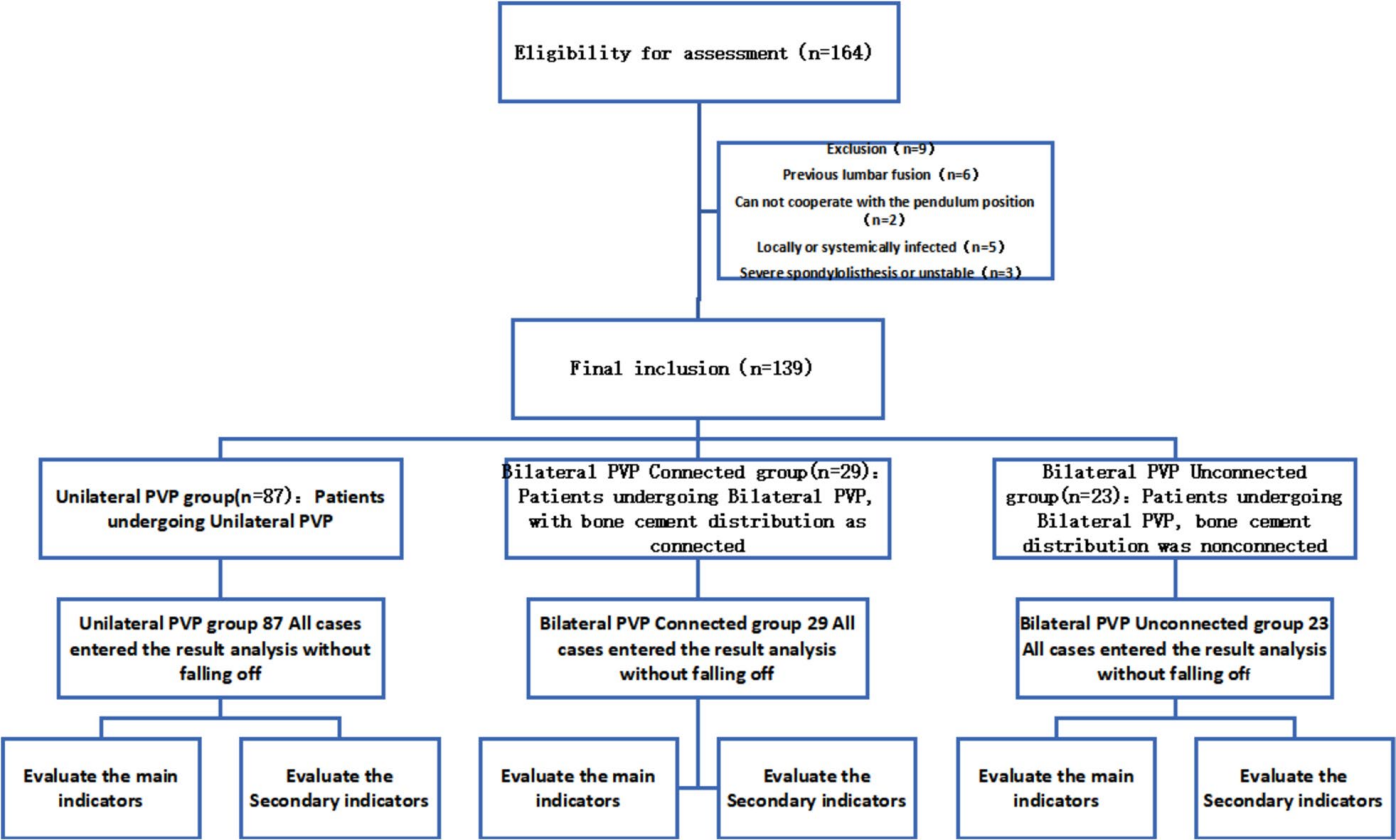
Flow chart of test grouping

The flow chart of the two groupings is shown in Fig. 3.

### Comparison of preoperative data between the two groups

Upon rigorous statistical scrutiny, it was ascertained that the tripartite groups demonstrated no statistically significant disparities concerning gender, age, BMI, BMD T value, PVP stage, historical medical records, preoperative VAS and ODI scores, initial vertebral body height, or the anterior–posterior kyphotic angle (*P* > 0.05). A granular breakdown of these findings can be gleaned from Table 4.

**Table 4:** Baseline characteristics

Items	Unilateral PVP group	Bilateral PVP Connected group	Bilateral PVP Unconnected group	F/X^2^	*P*
**Age ** $\overline{\mathrm{x}}\pm \mathrm{s }$	71.73 ± 8.08	72.31 ± 8.88	74.47 ± 8.45	0.990	0.374
**Sex(n/%)**				5.247	0.073
Male	27/31.00	10/34.50	2/8.70		
Female	60/69.00	19/65.50	21/91.30		
**BMI ** $\overline{\mathrm{x}}\pm \mathrm{s },\mathrm{ kg}/\mathrm{m}2$	24.90 ± 4.54	23.50 ± 3.61	24.98 ± 3.62	1.295	0.277
**BMD (T score) (n/%)**	-3.13 ± 1.00	-3.76 ± 0.83	-3.70 ± 0.80	3.197	0.202
-**2.5SD**	62/71.30	19/65.50	20/87.00		
** ≥ -2.5SD**	25/28.70	10/34.50	3/13.00		
**Fracture site(n/%)**				2.026	0.731
T5-T10	7/8.00	3/10.30	4/17.40		
T11-L2	62/71.30	19/65.50	15/65.20		
L3-L5	18/20.70	7/24.10	4/17.40		
**Cardiovascular and cerebrovascular (n/%)**	43/49.40	16/55.20	13/56.50	0.534	0.766
**Respiratory system(n/%)**	22/25.30	5/17.20	3/13.00	2.019	0.364
**Endocrine system(n/%)**	39/44.80	7/24.10	6/26.10	5.485	0.064
**Metabolic diseases(n/%)**	46/52.90	11/37.90	13/56.50	2.361	0.307
**History of smoking(n/%)**	11/12.60	7/24.10	1/4.30	4.464	0.107
**History of drinking(n/%)**	13/14.9	4/13.80	0/0.00	3.867	0.145
**Preoperative VAS, ** $\overline{\mathrm{x}}\pm \mathrm{s },\mathrm{ score}$	7.02 ± 1.38	7.55 ± 1.18	6.95 ± 1.49	1.845	0.162
**Preoperative ODI, ** $\overline{\mathrm{x}}\pm \mathrm{s },\mathrm{ \%}$	68.20 ± 9.45	69.37 ± 8.41	68.13 ± 10.22	0.185	0.832
**PFH ** $\overline{\mathrm{x}}\pm \mathrm{s },\mathrm{ cm}$	2.09 ± 0.23	2.12 ± 0.20	2.10 ± 0.19	0.221	0.802
**LKA ** $\overline{\mathrm{x}}\pm \mathrm{s }^\circ$	14.94 ± 0.82	15.19 ± 0.81	15.02 ± 0.77	1.080	0.342

The continuous value was given as the mean and the standard deviation. *BMI* Body mass index = weight/height^2^, *BMD* bone mineral density, *PFH* preoperative fractured vertebral body height, *LKA* local kyphosis angle, *VAS* visual analogue scale, *ODI* oswestry disability index

### Comparison of preoperative and postoperative VAS and ODI scores for low back pain between the two groups

**Fig. 4:**
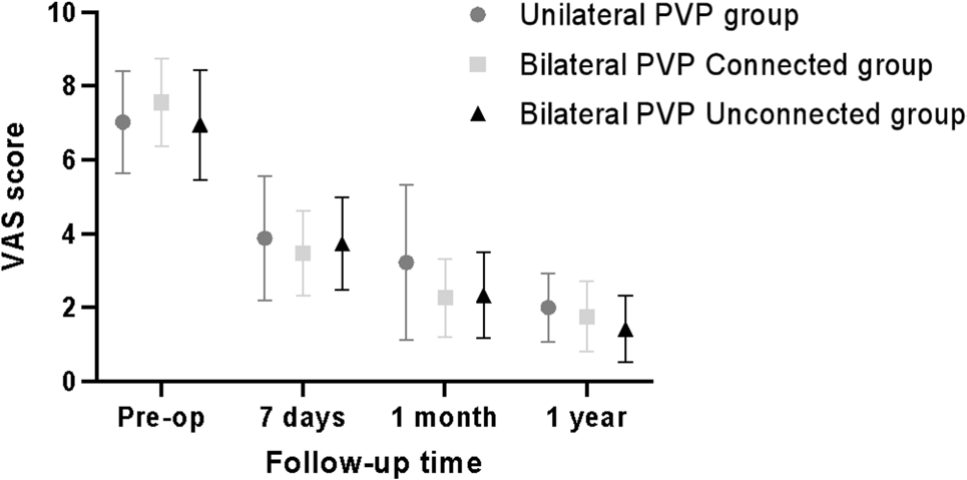
Comparison of VAS between the two groups

**Fig. 5:**
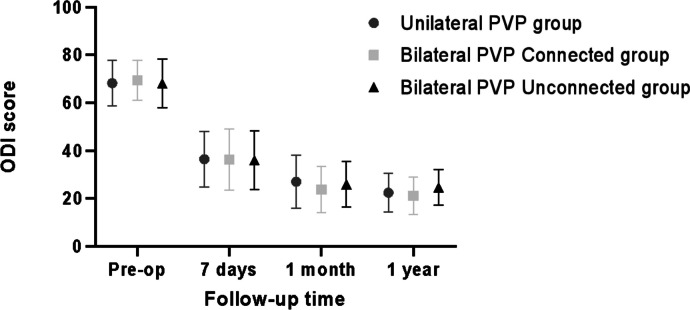
Comparison of ODI between the two groups

For the postoperative PVP assessments at specific timelines (7 days, 1 month, and 1 year), the bilateral PVP scores manifested no significant difference (*P* > 0.05). Nevertheless, the bilateral PVP outcomes were markedly superior when juxtaposed against the unilateral PVP results (*P* < 0.05). Such observations, detailed in Table 5 and illustrated in Figs. 4, 5, intimate a potentially more favorable long-term prognosis for the bilateral PVP cohort.

**Table 5:** Comparison of VAS and ODI scores for postoperative low back pain in the three groups

Items	follow-up time	Unilateral PVP group	Bilateral PVP Connected group	Bilateral PVP Unconnected group	F/X^2^	P
**Low back VAS score ** $\overline{\mathrm{x}}\pm \mathrm{s },\mathrm{ score}$	**1 week**	3.88 ± 1.68	3.48 ± 1.15	3.73 ± 1.25	0.542	0.467
	**1 month**	3.22 ± 2.09	2.27 ± 1.06	2.34 ± 1.15	3.623	**0.015**
	**1 year**	2.00 ± 0.94	1.75 ± 0.95	1.43 ± 0.89	4.145	**0.032**
**Low back ODI score ** $\overline{\mathrm{x}}\pm \mathrm{s },\mathrm{ \%}$	**1 week**	36.44 ± 11.70	36.27 ± 12.68	36.08 ± 12.27	0.009	0.991
	**1 month**	27.05 ± 11.09	23.79 ± 9.56	25.91 ± 9.52	1.051	0.353
	**1 year**	22.48 ± 8.12	21.20 ± 7.74	24.65 ± 7.38	1.226	0.297

The continuous value was given as the mean and the standard deviation. *VAS* visual analogue scale, *ODI* oswestry disability index

### Comparison of postoperative vertebral body height recovery rate, vertebral body local kyphosis angle, re-fracture and position of cement and endplate among the three groups

In the aftermath of the procedures, evaluations pertaining to the vertebral height recovery rate, local kyphotic angle alterations, and cement-to-endplate positioning in the bilateral PVP category yielded no significant variance (*P* > 0.05). However, the incidence of re-fractures—both in the surgically treated and adjacent vertebrae—was substantially attenuated in the bilateral PVP cohort when contrasted with the unilateral PVP group, and this difference achieved statistical significance (*P* < 0.05). A comprehensive tableau of these findings is presented in Table 6.

**Table 6:** comparative analysis of postoperative metrics—vertebral height recovery rate, local kyphosis angle, fracture incidence, and cement-endplate positioning—across the three study groups

Items	Unilateral PVP group	Bilateral PVP Connected group	Bilateral PVP Unconnected group	F/X^2^	P
PRH, 1 week $\overline{\mathrm{x}}\pm \mathrm{s },\mathrm{ cm }$	2.46 ± 0.13	2.47 ± 0.10	2.48 ± 0.08	0.414	0.662
LKA, 1 week $\overline{\mathrm{x}}\pm \mathrm{s }^\circ$	6.98 ± 0.38	7.06 ± 0.29	7.06 ± 0.31	0.898	0.410
Refracture of injured vertebrae and Adjacent vertebral fracture(n/%)	16/18.4	0/0.0	1/4.3	19.059	**0.015**
Position of bone cement and end plate(n/%)				3.868	0.114
Contact the upper and lower end plate	45/51.70	13/44.80	10/43.50		
Contact the upper or lower end plate	25/28.70	7/24.10	8/34.80		
The central vertebral bodies	17/19.50	9/31.00	5/21.70		

The continuous value was given as the mean and the standard deviation. *PRH* postoperative restored vertebral body height, *LKA* local kyphosis angle

### Comparison of secondary outcome indicators among the three groups

Upon examining the secondary outcome indicators, it was discerned that both the operative duration and the cumulative hospital expenses were substantially diminished in the Unilateral PVP cohort as compared to both the Bilateral PVP-Connected and Bilateral PVP-Unconnected cohorts. The disparities in these parameters were statistically significant (*P* < 0.000). However, when assessing the bone cement dosage and the incidence of bone cement leakage, no substantial variances were observed amongst the three cohorts (*P* > 0.05). Detailed statistics are presented in Table 7.

**Table 7:** Comparison of the secondary outcome measures

Items	Unilateral PVP group	Bilateral PVP Connected group	Bilateral PVP Unconnected group	F/X^2^	P
Operative time $\overline{\mathrm{x}}\pm \mathrm{s },\mathrm{ min}$	36.54 ± 8.49	42.75 ± 3.15	43.91 ± 5.83	14.028	**0.000**
Bone cement injection volume $\overline{\mathrm{x}}\pm \mathrm{s },\mathrm{ ml}$	5.70 ± 1.33	5.88 ± 1.04	5.98 ± 1.65	0.467	0.628
Cement leakage (n/%)	26/29.90	8/27.60	8/34.80	0.327	0.849
△Expenses $\overline{\mathrm{x}}\pm \mathrm{s },\mathrm{ RMB}$	11,645.39 ± 3236.34	13,326.05 ± 4331.18	13,171.93 ± 1921.54	3.832	**0.024**

The continuous value was given as the mean and the standard deviation

Stay. ∆ Results are presented in Chinese yuan

### Typical cases

See Fig. 6

**Fig. 6:**
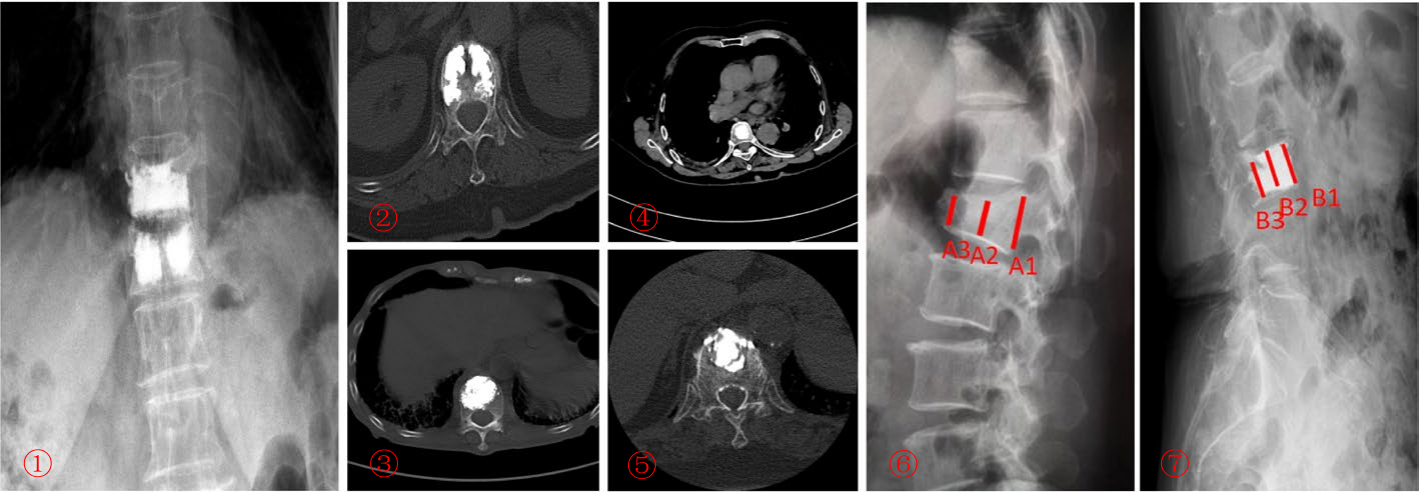
Postoperative x-ray and CT imaging. ① Bilateral PVP Disconnected group: Radiographic image (standing position).② Bilateral PVP Unconnected group: Computed Tomography (CT) scan (supine position). ③ Bilateral PVP Connecting group: CT scan (supine position). ④ Bilateral PVP Connected group: CT scan (supine position). ⑤ Unilateral PVP group: CT scan (supine position). ⑥ Preoperative vertebral height: Radiographic image wherein A1 denotes the posterior margin of the preoperative vertebra, A2 represents the height of the preoperative fracture, and A3 signifies the anterior margin of the preoperative vertebra. ⑦ PVP postoperative measured vertebral height: Radiographic image (standing position). B1 refers to the posterior margin of the vertebral body, B2 pertains to the height of the vertebral body, and B3 corresponds to the anterior boundary of the vertebral body

## Discussion

### Summary of evidence

With the progression of age among global populations, there is a noted increase in the incidence of osteoporotic vertebral compression fractures. Bilateral Percutaneous Vertebroplasty (PVP) and Unilateral Percutaneous Vertebroplasty are internationally endorsed as minimally invasive interventions designed for the swift recuperation of osteoporotic vertebral compression fractures [11]. Their applications have been extensively adopted for managing osteoporotic vertebral compression fractures within the thoracolumbar region, standing as testament to their clinical efficacy, and solidifying their status as a standard surgical recourse [12–14]. The therapeutic mechanism underlying these procedures involves the reinforcement of vertebral body integrity and stability through the infusion of bone cement [15]. Consequently, the distribution and quantity of bone cement within the vertebral structure have implications for clinical outcomes.

Historically, PVP made its debut in 1987, targeting cervical hemangiomas and thoracolumbar OVCFs [16]. This procedure involves the percutaneous administration of cement into the vertebral body via the pedicular space, aiming to stabilize the fractured mass, reconstitute bone architecture and mechanical resilience, thwart any further compression ensuing from the fracture, and alleviate pain. The cement, upon its introduction, solidifies trabecular bone by infiltrating the trabecular voids of the afflicted vertebrae, consequently redistributing trabecular pressure and enhancing skeletal robustness. Concurrently, the thermal aftermath of this process incapacitates the peripheral nerves within the vertebrae, culminating in pain reduction [17].

Two predominant modalities of PVP are recognized: unilateral and bilateral. The choice between these has been a topic of ongoing debate [18]. Some literature suggests that bilateral puncture facilitates superior cement filling due to its bidirectional application. However, counterarguments propose that unilateral puncture suffices for the surgical prerequisites. Concerns arise with bilateral puncture as it might inflict damage to the spine's weight-bearing structures, elongate operative durations, escalate medical expenditures, and augment patients' financial encumbrances. In contrast, unilateral PVP boasts advantages such as being less invasive, necessitating reduced intraoperative fluoroscopy, and offering expedited surgery durations [19]. This makes unilateral PVP particularly favorable for patients with limited surgical tolerance, like geriatric and frail individuals. Despite this, it's worth noting that the risk of cement leakage is heightened with the unilateral technique. On the other hand, bilateral PVP augments the homogeneous distribution of cement within the compromised vertebral body, a factor instrumental in alleviating pain and augmenting functional outcomes.

In a study spearheaded by Qi Zhenliang et al. [10], findings illustrated marked improvements in ODI and VAS post-surgery for patients undergoing both unilateral and bilateral PVP, when juxtaposed with their preoperative scores (*P* < 0.01). However, when comparing the postoperative ODI and VAS between the two groups, no substantial disparities were observed (*P* > O.05). Subsequent imaging underscored cement filling extending beyond the midline for all patients. This suggests that both unilateral and bilateral PVP have commendable efficacies in mitigating pain. Conversely, Zhang Huilin et al. [18] embarked on a retrospective analysis, revealing a decline in VAS and ODI scores at both 1 and 6 months post-surgery in both unilateral and bilateral PVP cohorts. This indicates a consistent therapeutic benefit and parallel clinical efficacy between the two surgical modalities [20]. A meta-analysis further demonstrated analogous ODI scores between unilateral and bilateral PVP [MD = 0.03, 95% CI (0.57,0.62), P = 0.93]; (*P* = 0.73, I^2^ = 0%). Our study corroborates these findings, emphasizing that ODI scores pertaining to low back pain, vertebral height recovery rates, and vertebral local lordosis angles are congruent across the three groups. Nevertheless, 1- and 12-month postoperative VAS scores for low back pain were noticeably lower in the bilateral PVP group compared to their unilateral counterparts. Additionally, the incidence of postoperative re-fractures was substantially elevated in the unilateral PVP group. This accentuates the notion that bilateral PVP enhances the uniform distribution of bone cement within the compromised vertebra, optimizing pain relief, functional improvement, and reducing the likelihood of recurrent fractures.

In the realm of procedural approaches, LOU ZHAO et al. [21] deduced that the unilateral PVP method may culminate in lopsided cement distribution or heterogeneous dispersion. Such unevenness could precipitate a collapse of the opposing side, thereby compromising vertebral stability. Such a predicament is less likely with the bilateral approach. Moreover, the bilateral strategy obviates the need for high puncture angles, potentially attenuating surgical trauma inflicted by less adept procedures [22]. However, another study contended that the bilateral approach augments the risk of radiation exposure, jeopardizing both patient and clinician safety [17, 23]. The bilateral approach might also amplify the likelihood of perivertebral tissue injuries. It's posited that during a unilateral pedicle approach, judiciously elevating the puncture angle could enable cement distribution to the contralateral side, preventing a cement imbalance and the ensuing collapse of the untreated side.

Bone cement leakage stands as a prevalent complication inherent to vertebroplasty. Such leakage can manifest symptoms of nerve irritation, particularly when it culminates in nerve root compression. Comparative analyses between unilateral and bilateral PVP showed an inconclusive difference in cement leakage rates (OR = 1.00, 95% CI [0.67,1.50], *P* = 1.00; *P* = 0.98, I^2^ = 0%) [24, 25]. Klazen et al. [26] in a comprehensive multicenter trial employing CT, noted a bone cement leakage rate reaching up to 72% in PVP procedures. This suggests that the PVP technique does not inherently augment the propensity for cement leakage. Parallel findings by Tao Li et al. [27] through a retrospective study indicated an equivalency in the mean volume of bone cement injected between the two procedural approaches. Furthermore, domestic scholars [28] employing a three-dimensional finite element analysis of PVP fillers, posited an optimal bone cement dosage of 4 ml, striking a balance between therapeutic efficacy and mitigation of risks such as adjacent vertebral fractures and cement leakages. It is worth noting that in our study, adjacent vertebral fractures were documented in both procedural groups (16 instances in the unilateral PVP group and 1 in the bilateral PVP group). This might be attributable to over-injection of bone cement. Moreover, the refracture rates for injured and adjacent vertebrae post-surgery reached a substantial 88.23%. Prior research indicated a post-PVP refracture incidence of approximately 11%, while our study documented a higher rate of 18.4%. This notable discrepancy might be attributed to the limited sample size considered in our analysis. Three separate meta-analyses [29–31] highlighted a discernible difference in operative durations between unilateral and bilateral approaches (MD = 8.42, 95% CI [13.17, 3.66], *P* = 0.0005; *P* < 0.00001, I^2^ = 98%). These findings dovetail with our results, underscoring that both operational durations and aggregate hospital expenses were markedly reduced in the unilateral PVP group when juxtaposed against both the bilateral PVP and bilateral PVP unconnected cohorts.

### Limitations of the article

(i) The present study is retrospective in nature. As such, there is an inherent risk of missing data or time-lagged data recordings, both of which could introduce biases into the results. However, the research team, to which the authors are affiliated, has meticulously and rigorously documented postoperative outcome measures across all groups, ensuring maximal data integrity and completeness. (ii) The sample size in this investigation is relatively modest, which potentially reduces the statistical power and might introduce reporting biases. (iii) Evaluation metrics were circumscribed. Pertinent indicators, such as detailed treatment costs and specific adverse outcomes, were not incorporated into the assessment. (iv) The surgical interventions were performed by various surgeons, which implies that the operational techniques might not have been perfectly standardized across the board. (v) Comprehensive assessments, such as pre-and post-operative vertebral body kyphosis Cobb angles, and evaluations of lifestyle factors like physical activity, tobacco use, and alcohol consumption, were not conducted. (vi) Given the ongoing debate regarding the impact of cement distribution on outcomes following percutaneous kyphoplasty or Percutaneous vertebroplasty, the findings of this study necessitate validation through more expansive, multicenter, prospective investigations. These subsequent studies would aim to better delineate the relationship between intravertebral cement distribution and PVP clinical outcomes.

### Conclusion

Relative to unilateral PVP, bilateral PVP presents a more favorable long-term prognostic outlook for patients suffering from vertebral compression fractures. Moreover, bilateral PVP markedly diminishes the risk of injury to the vertebrae. In contrast, the unilateral PVP cohort benefits from advantages such as reduced operative durations, minimal surgical invasiveness, and diminished overall hospital expenses, underscoring its clinical utility and value.

## Data Availability

Follow-up regarding the Effect of different cement distribution in bilateral and unilateral Percutaneous vertebro plasty on the clinical efficacy of vertebral compression fractures is not complete, so the dataset analyzed in this study is not publicly available but is available to the corresponding author on reasonable request. Follow-up of patients after Percutaneous vertebro plasty is incomplete, so the dataset analyzed in this study is not publicly available but is available to the corresponding author on reasonable request.

## Abbreviations

BMD: Bone mineral density
EOH: Estimated original vertebral body height
HRR: Height restoration rate
LKA: Local kyphosis angle
ODI: Oswestry disability index
OVCF: Osteoporotic vertebral compression fractures
PFH: Preoperative fractured vertebral body height
PKP: Percutaneous kyphoplasty
VAS: Visual analogue scale
PMMA: Polymethyl methacrylate
PRH: Postoperative restored vertebral body height
